# Polymers in Engineering Extracellular Vesicle Mimetics: Current Status and Prospective

**DOI:** 10.3390/pharmaceutics15051496

**Published:** 2023-05-14

**Authors:** Xinyue Wei, Sihang Liu, Yifeng Cao, Zhen Wang, Shengfu Chen

**Affiliations:** 1 Key Laboratory of Biomass Chemical Engineering of Ministry of Education, College of Chemical and Biological Engineering, Zhejiang University, Hangzhou 310027, China; 2 State Key Laboratory of Advanced Optical Communication Systems and Networks, Key Laboratory for Thin Film and Microfabrication of the Ministry of Education, UM-SJTU Joint Institute, Shanghai Jiao Tong University, Shanghai 200240, China; 3 Department of Electronic Chemicals, Institute of Zhejiang University-Quzhou, Quzhou 324000, China; 4 Zhejiang Sundoc Pharmaceutical Science and Tech Co., Ltd., Hangzhou 310051, China

**Keywords:** extracellular vesicle mimetics, extracellular vesicles, semi-synthetic, drug delivery systems, polymer modification, zwitterionic biomaterials, delivery efficiency, cancer treatment

## Abstract

The maintenance of a high delivery efficiency by traditional nanomedicines during cancer treatment is a challenging task. As a natural mediator for short-distance intercellular communication, extracellular vesicles (EVs) have garnered significant attention owing to their low immunogenicity and high targeting ability. They can load a variety of major drugs, thus offering immense potential. In order to overcome the limitations of EVs and establish them as an ideal drug delivery system, polymer-engineered extracellular vesicle mimics (EVMs) have been developed and applied in cancer therapy. In this review, we discuss the current status of polymer-based extracellular vesicle mimics in drug delivery, and analyze their structural and functional properties based on the design of an ideal drug carrier. We anticipate that this review will facilitate a deeper understanding of the extracellular vesicular mimetic drug delivery system, and stimulate the progress and advancement of this field.

## 1. Introduction

Cancer, ranking second only to heart disease as a leading cause of death worldwide, currently faces significant limitations in terms of effective treatment [1]. One promising avenue for improvement is using nanomedicines, which undergo a five-step process known as the “CAPIR” (Circulation, Accumulation, Penetration, Internalization, and Release) cascade to reach and treat tumor sites. During their blood circulation, nanomedicines must remain structurally stable in the physiological environment while avoiding detection by the immune system as they travel through the bloodstream to the tumor site. The next step is accumulation, wherein nanomedicines aggregate at the tumor tissue. This is caused by the enhanced permeability and retention (EPR) effect, taking advantage of the increased vascular permeability and absence of a lymphatic drainage system in tumor tissues. Nanopharmaceuticals with a particle size of 100 nm have been shown to be more efficient in earlier studies. The third step requires nanomedicines to penetrate tumor tissue. After accumulating in the tumor tissue, nanomedicines must penetrate through the extracellular matrix between tumor cells to attack distant and deep tumor cells. Smaller nanoparticles are generally believed to have an advantage in this process, with those over 100 nm exhibiting relatively weak penetration. In the fourth step, nanomedicines need to bind to the surface of tumor cells and be internalized to achieve better efficiency in killing them. This can be achieved by immobilizing ligands targeting tumor-specific targets on the nanoparticles or by utilizing differences in tumor tissue pH and redox conditions compared to healthy cells. Finally, the fifth step involves releasing chemotherapeutic drugs inside tumor cells. The nanomedicine’s stability needs to be destroyed to achieve this release.

However, the five cascades are filled with conflicting performance requirements for nanomedicines. In blood circulation, for example, nanomedicines need to remain superficially inert to avoid recognition by the immune system, but need to be actively de-inert to bind to cells in the process of internalization; 100 nm nanomedicines can accumulate efficiently in tumor tissue, but cannot penetrate the extracellular matrix. These contradictory characteristics make it seem impossible to realize delivery efficiency maximization in every link at the same time, even if complex intelligent structures have been designed [2].

Natural extracellular vesicles (EVs) are a crucial means of short-distance communication between cells and possess organotropism abilities. For instance, tumor cell-secreted EVs tend to accumulate within the same tumor tissue after entering the bloodstream. Organotropism is driven by the diverse surface proteins expressed by EVs that prevent their recognition by the immune system as carriers of foreign antigens, enabling them to achieve short-distance delivery and target specific cells before eventually being internalized and decomposed [3,4,5]. Thus, natural EVs can intelligently regulate the conflicting needs of CAPIR cascades, making them an exciting prospect for cancer treatment. Consequently, some EV-based therapies are currently under clinical trials [6].

**Figure 1:**
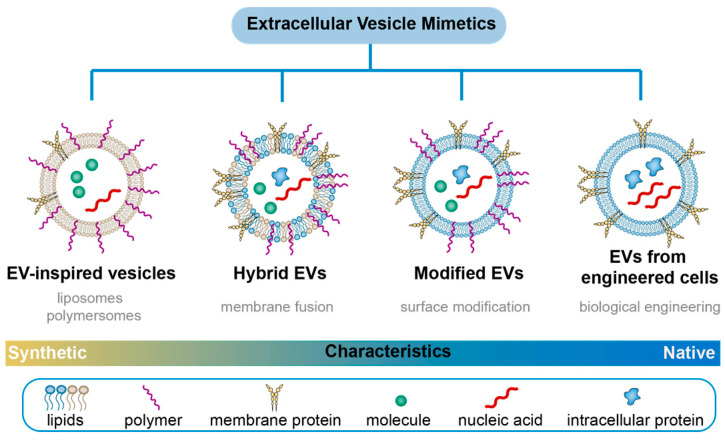
Extracellular vesicle mimetics (EVMs) landscape: the classification of EVMs from synthetic approaches.

Although extracellular vesicles (EVs) are cell secretions, they undergo specific metabolic pathways that lead to their enrichment in metabolic organs such as the liver, kidney, and spleen. This enrichment is not conducive to long blood circulation, which is necessary for EVs to function effectively as a nano-drug delivery system. Furthermore, the diverse ligand proteins expressed on the surface of natural EVs make them susceptible to capture by non-target cells with corresponding receptors, leading to inefficient drug delivery [7,8,9]. While local injection of EVs around the lesion site or their sustained release via hydrogel entrapment can mitigate the effects of blood circulation, local administration requires invasive means near the tumor tissue and provides limited effect in patients with distal metastatic symptoms [10,11]. Therefore, the use of a nano-drug delivery system through the bloodstream remains the preferred choice for drug targeting. Recent advances in designing nano-drug carriers with natural and synthetic polymers have led to the development of functional polymer-based extracellular vesicle mimics (EVMs) [12,13]. These EVMs preserve the advantages of both natural EVs and polymers, such as improved targeting and extended blood circulation time [14,15]. In this review, we focus on recent advances in polymer-based EVMs based on their structural characteristics. Note that unless otherwise specified, the EVMs discussed in this paper have a particle size of 40–100 nm (Figure 1).

## 2. Structure Design of Extracellular Vesicle Mimetics

### 2.1. Structure of Nanosized Drug-Loaded EVs

Exosomes containing nanomedicine have a three-part structure that includes cargo [16,17], lipid membrane [18,19,20], and surface ligands [21,22] (Table 1). These extracellular vesicles (EVs) are capable of transporting a variety of drugs, with hydrophilic drugs embedded in the hydrophilic core and hydrophobic drugs anchored in the lipid bilayer. This makes EVs an ideal vehicle for delivering most drugs, including nucleic acids, lipids, and proteins. While the lipid types in EVs are determined by their source, the proportions of different lipids can vary significantly from those found in their source cells (e.g., glycolipids, phosphatidylserine). Importantly, studies have shown that all EVs originating from five different cell types (B16BL6 murine melanoma cells, C2C12 murine myoblast cells, NIH3T3 murine fibroblast cells, MAEC murine aortic endothelial cells, and RAW264.7 murine macrophage-like cells) are rapidly cleared from systemic circulation within minutes due to liver clearance [23]. This suggests that there must be universal components present in all EVs that lead to rapid identification and clearance. Indeed, when the phosphatidylserine (PS)-binding sites on macrophages were blocked using PS-rich liposomes, the clearance rate of EVs in the blood decreased significantly. Therefore, the presence of PS is an important factor influencing the clearance of EVs [24,25].

**Table 1:** Components and characteristics of EVs from different cell origins.

Cells Origin	Components	Characteristics	Ref.
erythrocytes	Cargo: ALIX, HSP-70, TSG101, syntenin, hemoglobin, acetylcholinesterase, etc.; Lipid: phosphatidylserine, phosphatidylethanolamine, phosphatidylinositides, sphingomyelin, Surface display: CD47, flotillin, CD63, CD235a, CR1, CD59, etc.	no nuclear and mitochondrial DNA; preventing immune clearance pathways; prolonged circulation time; conveniently accessing from human blood; low cost for expansion	[26,27,28,29,30]
macrophages	Cargo: syntenin ① produced from activated macrophages: CXCL2, IL-17, TNF-α, CCL3, CXCL10, miR-155; ② produced from nonclassical macrophages: miR-21-5p, miR-155-5p Surface display: CD63, CD9, CD81, etc.	induced inflammatory conditions; inhibiting migration; regulated tumor cells’ proliferation, invasion, and angiogenesis	[31,32,33]
natural killer cells	Cargo: perforin, granulysin, etc.; Surface display: CD63, FasL, granzyme A, granzyme B, etc.	immuno-surveillance, host defense against cancer and pathogen infections; increased the proliferation rate of NK cells	[34,35,36,37]
dendritic cells	Cargo: Hsp70, Mart-1 peptides, annexins, RAB proteins, TSG101, etc.; Surface display: ① MHC class I- and class II-peptide complexes; ② CD40, CD80, CD86, CD8, ICAM, TNF-α, TRAIL, NKG2D ligands, etc.	Secreted from immature DC failed to induce potent T-cell responses; modulating the antigen-specific response; activating NK cells; induced Treg cell differentiation; boosting strategies of immunotherapy	[38,39,40]
T lymphocytes	Cargo: miR-298-5p, miR-150, syntenin, etc.; Surface display: CD3, CD2, CD4, CD8, CD11c, CD25, CD69, LFA-1, CXCR4, FasL, GITR, TNF-α, PD-1, etc.	mediating depletion of MSCs and CAFs; killing tumor-derived MSCs; decreased tumor cells’ metastasis; restored the tumor microenvironment.	[41,42,43]
mesenchymal stem cells	Cargo: PLP2, TIMP-1, TIMP-2, miR-21, miR-34a, syntenin, etc. Lipid: acyl carnitines, lysophosphatidylcholines, cholesterol esters, cardiolipins, phosphatidylserine, phosphatidylglycerols, phosphatidylinositols, phosphatidylethanolamines, etc.; Surface display: CD63, CD81, CD9, CD29, CD44, CD73, CD47, CD90, PDGFR, LAMP2, etc.	alleviated inflammatory response	[44,45,46,47]
tumor cells	Cargo: Hsp90, Hsp70, annexin, MMPs, etc.; Lipid: cholesterol esters, sphingomyelin, phosphatidylserine, cardiolipins, phosphatidylglycerols, phosphatidylinositols, phatidylethanolamines, etc.; Surface display: ① MHC class I- and class II-peptide complexes, intra-exosomal and membrane-bound antigens; ② CD9, CD63, CD37, CD81, CD82, CD53, LFA1, MFGE8, TIM1/4, PD-L1, integrins, etc.	inhibition of tumor proliferation	[47,48,49,50,51]
plant cells	Cargo: miR-168c, ath-miR167a, etc.; Lipid: phosphatidic acids, phosphatidylethanolamine, phosphatidylcholine, phosphatydilinositol phosphate, digalactoyldiacylglycerol, shogaol, etc.; Surface display: CD63, CD9, CD81, etc.	grape-derived EVs: accelerating organoid structure formation; ginger-derived EVs: a reduced sign of inflammation and local lymphocytic infiltration; broccoli-derived EVs: protecting against the development of colitis	[52]

Diverse surface ligands on extracellular vesicles (EVs) offer a myriad of possibilities for drug delivery. A broad range of proteins, including lysosomal-associated membrane proteins (LAMP), integrins, proteoglycans, and tetraspanin proteins, may be integrated into or attached to the membranes of EVs, thereby altering their functionality. Highly expressed surface proteins such as CD47, CD55, and CD59 can prolong the circulation time of EVs by evading the immune system [53]. Binding integrins, tetrapeptides (CD151, CD63, Tspan8, etc.), and other proteins (fibronectin, Wnt4, etc.) to EV surfaces can modulate their accumulation in specific organs [53]. For instance, the presence of α6β4 and α6β1 integrins has been linked to lung metastasis, while αvβ5 is related to liver metastasis [54]. EVs containing Tspan8 tend to accumulate in the pancreas, and those expressing CD9, CD63, CD81, and Alix can cross the blood–brain barrier in transwell experiments [55]. Moreover, polymer-engineered EV membrane-derived vesicles (EVMs) can express desirable ligands based on the application scenarios, further enhancing the efficiency of drug delivery.

### 2.2. Preparation of Polymer Engineering EVMs

EVMs can be broadly categorized into three groups. The first group is surface-modified EVMs (Sur-EVMs), which retain the natural membrane structure of EVs but undergo further modifications through synthetic biology or chemical methods, enhancing their long blood circulation and targeting ability towards specific cells. The second group is composed of natural EV-inspired fully synthesized EVMs (Syn-EVMs), featuring a clear physicochemical structure and properties with a greater controllable surface modification degree and industrial production stability. The third group consists of hybrid EVMs (Hyb-EVMs), which combine the advantages of Syn-EVMs and Sur-EVMs through membrane fusion.

#### 2.2.1. Surface-Modified EVs (Sur-EVMs)

**Figure 2:**
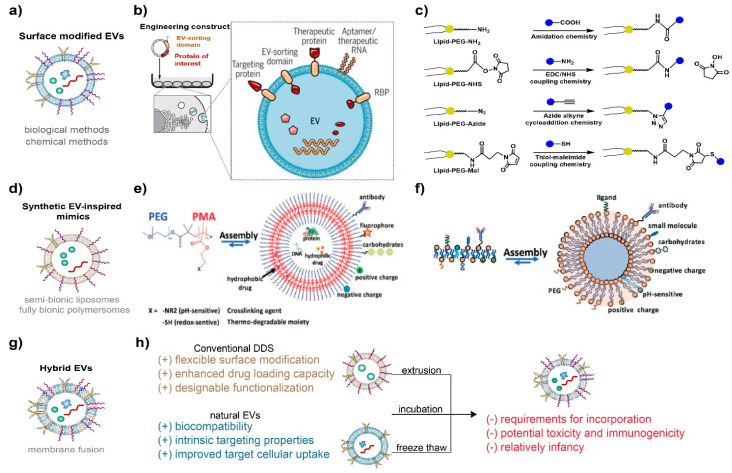
(**a**) Schematic of engineered EVMs by surface modification. (**b**) Illustration of the biological functionalization strategy by genetically engineering. Reproduced with permission from ref. [56]. Copyright 2019, AAAS. (**c**) Schematic showing different facile chemistries for surface functionalization of EVs. Reproduced with permission from ref. [57]. Copyright 2020, Royal Society of Chemistry. (**d**) Schematic of EV-inspired vesicles by full synthesis. (**e**) Schematic of liposome self-assembly from lipids. The composition of lipids can be engineered (charge, pH-sensitive) and/or prefunctionalized with different molecules (antibodies, proteins, carbohydrates, PEG, and other ones) to confer new properties (specific targeting, etc.). Reproduced with permission from ref. [89]. Copyright 2020, John Wiley and Sons. (**f**) Schematic representation of polymersome self-assembly from a representative PEG-b-PMA copolymer. The polymersomes can be modified at their surface (biomolecules, fluorophores, and charged groups by physi-/chemisorption), at the inner layer of the membrane (cross-linking, pH- and redox-sensitive moieties, indicated as X), in terms of loading in the aqueous core and in the hydrophobic part of the multilayer. Reproduced with permission from ref. [89]. Copyright 2020, John Wiley and Sons. (**g**) Schematic of hybrid EVs by membrane fusion. (**h**) Illustration of advantages and limitations of hybrid EVs.

The surface modification of Sur-EVMs primarily employs synthetic biological methods [56] and chemical methods [57,58] (Figure 2a). Through genetic engineering, donor cells are transfected with plasmids expressing peptides or proteins to alter the surface characteristics of EVs (Figure 2b). For instance, CD47-modified EVs (ExosCD47), generated by transfecting donor cells with the corresponding plasmid [59], can effectively evade phagocytosis by the mononuclear phagocyte system (MPS) and prolong their blood circulation time [59]. Streptavidin-lactadherin-expressing Sur-EVMs (SAV-LA) can be prepared by transfecting donor cells with a plasmid vector encoding a fusion SAV-LA protein [60]. By combining EVMs with biotinylated immunostimulatory CpG DNA, CpG DNA-modified EVMs can be delivered effectively to DC cells with enhanced tumor antigen presentation capacity. Lydia et al. [61] engineered dendritic cells to express Lamp2b and fused them to the neuron-specific RVG peptide through transfection with Lamp-2b modified pEGFP-C1 vector. RVG-targeted EVMs delivered siRNA specifically to neurons, microglia, and oligodendrocytes in the brain, resulting in specific gene knockdown.

Biomolecules, including targeted peptides, proteins, and aptamers, as well as synthetic polymers such as nonionic polymers (e.g., PEG) and zwitterionic polymers (e.g., pCBMA), can be immobilized on the surface of EVs through chemical methods such as thiol-maleimide coupling chemistry, EDC/NHS coupling chemistry, azide-alkyne cycloaddition chemistry, and amidation chemistry [57] (Figure 2c). Gai et al. [62] utilized functional modified lipids and performed antibody coupling via bio-orthogonal copper-free click chemistry. They successfully coupled azide-modified anti-mouse CD11c-antibodies to the surfaces of EVMs, which strongly bound to DC cells. Lathwal et al. [63] engineered DNA tethers embedded in EVMs using atom transfer radical polymerization (ATRP) to achieve rapid and on-demand functionalization. Poly(oligo(ethylene oxide) methacrylate) (p(OEOMA)) and poly(carboxybetaine meth-acrylate) (pCBMA) were engineered onto the EVM membranes by ATRP to achieve lower immune recognition. Natural EVs were quickly cleared from the blood in about three hours, while 10% of Exo-pOEOMA and 24% of Exo-pCBMA hybrids remained in circulation even after 12 h. It is important to note that zwitterionic polymers, which are also electrically neutral, provide not only a more reliable adsorption barrier against nonspecific proteins but also excellent stability in the physiological environment. Therefore, zwitterions will be a key candidate in drug carrier designs [64,65]. The ionic hydration layer of the zwitterionic polymer is stronger than the hydrogen-bonded hydration layer of the nonionic polymer, and the enthalpy change required for nonspecific adsorption of proteins is much higher [66,67,68]. This inhibits the spontaneous adsorption of proteins on the material surface and decreases recognition by the reticular endothelial system, which avoids rapid clearance by the mononuclear phagocyte system from the blood.

Despite the advantages of natural EVs, Sur-EVMs face challenges in achieving high-quality preparation due to the heterogeneity caused by size, components, and cellular origin. Uneven invagination of the limiting membrane can result in size inequality and distinct total contents of fluid and solids. Additionally, different cell types and biological origins can lead to variations in surface proteins and inner cargoes [69,70,71].

#### 2.2.2. Synthetic EV-Inspired Mimics (Syn-EVMs)

Syn-EVMs have a clear structure that can be customized for specific drugs and target cells, as illustrated in Figure 2d. There are various methods available to prepare EV-inspired mimics, such as semi-bionic liposomes and fully bionic polymer vesicles. These EVMs mainly consist of lipids and amphiphilic block copolymers, which mimic the amphiphilicity of natural phospholipids and self-assemble into vesicles in solution.

Polymersomes offer higher chemical and physical stability than lipid-based liposomes due to the low entropy of mixing of polymers. This makes them an advantageous choice for creating stable EV-inspired mimics with improved blood circulation efficacy. The commonly used amphiphilic copolymers typically have hydrophilic blocks such as PEG, poly(ethylene oxide) (PEO), poly(2-methyl oxazoline) (PMOXA), poly(acrylic acid) (PAA), or poly[l-isocyanoalanine(2-thiophen-3-yl-ethyl)amide] (PIAT), and a hydrophobic block such as polystyrene(PS), poly(butadiene) (PB), or poly(lactic-co-glycolic acid) (PLGA) [72]. Membrane thickness plays a crucial role in the assembly’s stability, as shown in Figure 2e. The mechanical properties of polymersomes depend largely on the copolymer type and the length of the hydrophobic block. However, the metabolic pathway of polymersomes in vivo requires further study, which introduces uncertainties and risks in the clinical drug approval process for drugs using polymersomes as a delivery system. Additionally, while polymersomes have clear surface modification sites and controllable modification advantages, their main structure lacks flexibility. Therefore, replacing certain components often necessitates redesigning the overall structure.

Liposomes, which are self-assembled by lipid bilayer layers, offer a clear metabolic pathway and greater flexibility of local structural adjustment, making them more suitable for clinical drug development. Liposomes consist of amphiphilic lipids that form bilayers cocooning an aqueous interior from the external bulk aqueous phase [73] (Figure 2f). The non-polar hydrophobic lipid tails are stabilized by van der Waals forces, while the hydrophilic head groups interact with the aqueous phase. Semi-bionic liposomes can be prepared using the “top-down” method, which involves downsizing cell membranes or EVs through a filter membrane of appropriate size. Alternatively, the “bottom-up” method can be used to create liposomes by selecting specific lipids and gradually fusing them into a particular size [74]. Compared to the top-down approach, the bottom-up method enables more precise control of the liposome structure and produces a more definite surface modification effect, making it more conducive to the industrialization of liposome drugs [75,76,77]. However, liposome instability is a crucial issue when preparing clinical drugs. Due to the fluidity of the phospholipid membrane, liposomes tend to aggregate and fuse. Polysaccharide-coated liposomes have been found to reduce phospholipid fluidity, improve membrane bending rigidity, and enhance liposome stability. Chitosan and its derivatives are the most commonly used polysaccharides. Chitosan binds to the head molecule of the phospholipid membrane through electrostatic action via two methods: (1) incubating the liposome in chitosan solution; and (2) the inverse phase method by mixing the phospholipid molecule with chitosan. The former results in chitosan coating only on the outer layer of the liposome, while in the latter, the coating is present in both inner and outer layers [78]. Results indicate that the chitosan coated by incubation is highly heterogeneous, while those prepared by the inverse phase method have better distribution and higher bending rigidity [78,79]. Additionally, polysaccharides such as hyaluronic acid, sodium alginate, pectin, etc., have been used to coat liposomes, significantly improving their intestinal adhesion or tumor targeting [80].

Like Sur-EVMs, semi-bionic liposomes and full-bionic polymersomes also require surface modification with antifouling polymers (such as PEG or zwitterionic polymers) or target units (such as stimulating sensitive units and target proteins). Despite having a more well-defined structure and better physicochemical properties, achieving a delivery efficiency equivalent to that of natural EVs remains challenging for Syn-EVMs. For instance, nucleic acids encapsulated in liposomes may not be transcribed as efficiently as natural EVs after their delivery into targeted cells [73,81]. Hence, further exploration is necessary in the design of synthetic EV-inspired mimics.

#### 2.2.3. Hybrid EVMs (Hyb-EVMs)

Despite the advantages exhibited by Sur-EVMs and Syn-EVMs as drug-delivery carriers, both types of EVMs still have limitations that hinder their application. A new generation of delivery system, termed hybrid EVs, has been developed to overcome these limitations, including modification efficiency, structural stability, and delivery efficacy. Currently, three main membrane fusion methods, namely incubation, freeze–thaw, and extrusion, are used to prepare hybrid EVs (as shown in Figure 2g,h).

The simple incubation of liposomes with EVs may be sufficient to induce fusion, but this method is slow and dependent on temperature due to its direct impact on fusion kinetics [82]. Piffoux et al. [83] developed a faster temperature-dependent method using PEG to facilitate membrane fusion of EVs with synthetic cargo-carrying liposomes, enabling the transfer of lipophilic drugs with minimal loss (<10%) and significant yet imperfect transfer of hydrophilic drugs (approximately 50%). Cheng et al. [84] designed hGLV, a hybrid therapeutic nanovesicle, by fusing gene-engineered EVs overexpressing CD47 with drug-loaded thermosensitive liposomes, using a freeze–thaw method. The resulting hGLV exhibited long blood circulation from the CD47-overexpressing EVs and excellent photothermal therapy under laser irradiation from the thermosensitive liposomes [84]. Sun et al. [85] designed a clodronate (CLD)-loaded liposome and fibroblast-derived EV hybrid drug delivery system using the extrusion method. The CLD-loaded liposome depleted macrophages via apoptosis once they were recognized and engulfed by Kupffer cells in the liver, diminishing hepatic uptake and improving target delivery with the capacity for natural EV organotropism to fibroblasts.

Several other membrane fusion methods have been developed. Virus-simulating vesicles can fuse with macrophage-derived EVs to form hybrid EVs that have been used to deliver the CRISPR-Cas9 system for targeted gene editing [86]. These hybrid EVs were synthesized using a simple thin-film hydration followed by a membrane extrusion method, overcoming the limitations of poor yield and functional property loss in EV isolation. During EV and liposome fusions, EV-EV and liposome-liposome fusion are not expected. Ducrot et al. [87] developed an elegant process that uses electrostatic interactions to favor directed EV-liposome interactions using pH-sensitive lipids. At low pH, the pH-sensitive liposomes become positively charged, inducing specific EV-liposome electrostatic interactions, while EVs are still repulsed from one another (both negatively charged) and liposomes (both positively charged). Once fused, the hybrids become neutral and do not interact with each other, thereby controlling the number of fusion events per EV or liposome. Finally, the pH returns to neutral, and the hybrids regain a slightly negative zeta potential suitable for intravenous injection.

Hybrid EVs bearing both the natural characteristics of EVs and the designable structures and properties of synthetic materials show great promise for disease treatment. However, research on Hyb-EVMs in disease treatment is still in its infancy. Further understanding is required on how the incorporation of synthetic materials or processing procedures would alter the properties of EVs. Additionally, the potential toxicity and risk of immunogenicity of these Hyb-EVM systems should be thoroughly investigated [88].

### 2.3. Cargos and Cargo Loading Methods

Loading endogenous and exogenous cargo is a crucial process in engineering EVs for drug delivery. Due to their relatively high capacity for loading small molecules, proteins, and nucleic acids, EVMs provide an opportunity for efficient delivery of cargo to target cells. However, delivering bioactive macromolecules such as nucleic acids and proteins requires a safe and efficient drug delivery system due to their large molecular weight and complex active conformation [61,90]. The ability of EVMs to transport biological macromolecules mimicking natural EVs makes them an alternative to synthetic nanoparticles based on cationic lipids or polymers. To achieve better effectiveness in cancer treatment, it is important to improve the loading efficiency of cargo. Cargo loading can be performed either pre- or post-production of EVMs. Using appropriate agents or genetic-engineering-edited producers to secrete EVMs with target cargos in one step maintains the integrity and natural characteristics of the membrane [91,92,93]. Sinoporphyrin sodium [94], mCherry proteins [91], and RNA [92] can be loaded into EVs using this “pre-loading” approach; however, it presents challenges for sorting and separating EVMs due to uncontrollable drug loading. Post-loading is an efficient strategy for unambiguous cargo loading of EVMs. In addition to the simple method of incubating drugs with EVMs [93,95], electroporation [61,96], sonication [59,97], freeze and thaw cycles [98], and chemical transfection [99,100] can bypass the membrane for loading. Catalase was loaded into EVMs (exoCAT) ex vivo using the following approaches separately: incubation at room temperature, freeze–thaw cycles, and sonication [101]. The amount of catalase loaded into EVMs increased in the following order: incubation at RT < freeze/thaw cycle < sonication. ExoCAT obtained by sonication showed the highest catalytic activity, followed by exoCAT obtained by freeze–thaw cycles, and then incubation at room temperature. For gene loading, CRISPR-Cas9 was loaded into EVs using electroporation [102] and chemical transfection [82].

Chemically coupled EVMs can be loaded with various functional cargos using “lipid chemical reactions” or “membrane protein chemical reactions.” This technology enables controllable and adjustable multifunctional transformation of EVMs. Chemical coupling allows for the control of both the “loading quantity” and “loading site” of functional molecules on EVs, which makes it possible to achieve the “multiple loading” of multiple functional molecules for targeted delivery, drug tracing, and more. Nie et al. [103] developed a universal responsive EV-nano-bioconjugate platform. They used azide-modified exosomes from M1 polarized macrophages to conjugate with dibenzocyclooctyne-modified anti-CD47 antibodies and anti-signal regulatory protein alpha antibodies linked with pH-sensitive benzoic-imine bonds. In the tumor microenvironment, both antibodies were released and inhibited the CD47-SIRPα pathway of tumor cells, eliminating their immune escape under macrophage recognition. Additionally, EVs from M1 macrophages promoted the M1 polarization of macrophages and enabled them to attack tumor cells. The perfect synergism of M1 EVs and antibodies resulted in potent anticancer efficacy with minor side effects. In addition to delivering antibodies, researchers have found that EVs can modify the efficacy of antibody-drug conjugates (ADCs) by delivering them to both local and distant cancerous and non-cancerous cells. Based on antibody-antigen interactions, ADCs can bind to EVs secreted by cancer cells expressing the ADC target proteins [104].

## 3. Recent Advances of EVMs-Based Nanomedicines for Cancer Therapy

Due to the diversity of cargoes and inherent targetability of extracellular vesicles (EVs), their potential in drug delivery has garnered significant attention. Drug-loaded EVs have been extensively studied for cancer treatment after being engineered to meet delivery requirements. As previously mentioned, polymers such as PEG, proteins, peptides, and aptamers have been employed to engineer EVs and enhance therapeutic efficiency while meeting delivery requirements for cancer treatment (see Table 2). Depending on the active pharmaceutical ingredient, the use of EVs as a drug delivery carrier can be classified into small molecule chemotherapeutics, nucleic acids, and proteins.

### 3.1. EVM Delivery of Small Molecule Chemotherapeutics

Chemotherapeutic drugs are commonly used in clinical settings. However, since chemotherapeutic drugs vary in their hydrophobicity, the design of drug carriers must be tailored to their specific characteristics. Extracellular vesicles (EVs), on the other hand, can serve as a universal delivery platform for small-molecule chemotherapeutic drugs due to their ability to transport both hydrophobic and hydrophilic drugs. Negrea et al. [105] encapsulated doxorubicin (DOX), a typical hydrophobic chemotherapeutic drug for cancer therapy, into PEG-coated extracellular vesicles via incubation to target melanoma cells. This resulted in a weakened invasion ability of primary melanoma. Zhao et al. [106] developed a specific M1 macrophage-derived exosome-based drug delivery system to encapsulate gemcitabine (GEM), a hydrophilic deoxycytidine analogue. They achieved efficient delivery of the hydrophilic drug and sensitized GEM-resistant pancreatic cancer cells to chemotherapy by co-delivering Deferasirox, an oral iron chelator.

Developing efficient loading methods, improving scaled production, obtaining favorable pharmacokinetic properties of drugs in vivo (especially enhancing targeting ability and overcoming physiological barriers), and achieving responsive release are key objectives for extracellular vesicle (EVs)-based chemotherapy. Several endogenous and exogenous techniques can be used for loading cargoes, including sonication, electroporation, extrusion, and freeze–thawing [107]. Geng et al. [108] prepared engineered EVs via hybridization with pH-sensitive liposomes. DOX-loaded EVs prepared by simple Ca2+-mediated fusion showed better capacity for circumventing endosome entrapment than those produced through freeze–thaw and PEG8000-mediated fusion. Moreover, crossing the blood–brain barrier (BBB) presents a significant challenge for drugs targeting the brain. Zhu et al. [109] generated Angiopep-2 (Ang) and trans-activator of transcription peptides (TAT)-modified EVs, and loaded DOX through electroporation for glioma treatment. The high affinity between Ang and low-density lipoprotein receptor-related protein-1 enabled EVs to cross the BBB, and the cell-penetrating ability of TAT breached the blood-brain tumor barrier (BBTB), thus improving the efficacy of chemotherapeutic molecules for brain tumors.

Researchers have developed novel combinations of chemotherapy with extracellular vesicles (EVs) inspired by their natural benefits. Recently, a combined “eat me/don’t eat me” strategy was developed to achieve mononuclear phagocyte system (MPS) escape and efficient drug delivery. Cationized mannan-modified EVs derived from DC2.4 cells were administered to saturate the MPS (eat me strategy). Then, nanocarriers fused to CD47-enriched EVs originating from human serum were administered to avoid phagocytosis by the MPS (don’t eat me strategy). The nanocarriers were also loaded with DOX and functionalized with a novel organotropism peptide to promote tumor tissue accumulation and cancer cell uptake (eat me strategy) [110]. This study sheds light on overcoming phagocytic evasion and provides a strategy for significantly improving therapeutic outcomes, potentially enabling active drug delivery via targeted nanomedicines.

### 3.2. EVM Delivery of Nucleic Acid

With the promotion of COVID-19 mRNA vaccines, nucleic acid drugs are expected to become the third-largest type of drugs after small molecular drugs and antibody drugs. Due to their ability to carry genes, natural extracellular vesicles (EVs) are considered potential gene delivery systems for cancer therapy. Currently, lipid nanoparticles (LNPs) and adeno-associated virus vectors (AAV vectors) are the most commonly used vectors for nucleic acid delivery. However, non-liver tissue targeting and large-scale production limit the application of nucleic acid delivery in LNPs, and pre-existing antibodies against AAV capsids exclude many patients. As natural mediators in the body, EVs encapsulated with nucleic acids can improve targeting efficiency through surface-engineered ligands and reduce systemic toxicity due to their low immunogenicity. Murphy et al. [111] demonstrated that EVs exhibit higher RNA delivery efficiency than synthetic RNA delivery systems. However, the reported activation level of RNA loaded in EVs is much lower than that required for similar activation levels by the most advanced synthetic therapeutic RNA delivery system.

Cationic polymers such as poly(ethyleneimine) (PEI) and chitosan can bind to negatively charged nucleic acid through electrostatic interaction and condense into a small, compact structure. Combining cationic polymers with extracellular vesicles (EVMs) can improve the drug loading efficiency of nucleic acids [112]. A ternary complex system consisting of pDNA, PEI, and chondroitin sulfate has low cytotoxicity and a durable high expression efficiency, resulting in a higher number of ESAT-6 epitopes in EVs [113]. Additionally, EV-modified PEI/siRNA complexes showed improved physical and biological properties, inhibiting prostate carcinoma xenografts in vivo [114]. Diomede et al. [115] compared human periodontal-ligament stem cells (hPDLSCs) enriched with EVs to PEI-engineered EVs (PEI-EVs). The results suggested that PEI-EVs participate in the activation of the osteogenic process. Similar results were also found in biodegradable chitosan-based EVM systems [116,117]. Furthermore, Alshamsan et al. [118] found that hydrophobically modified PEI increased gene condensation compared to parent PEI, providing a more productive strategy for loading RNA cargo onto extracellular vesicles. This idea can also be applied to EVM-based gene delivery because the hydrophobically modified gene could improve stability and promote cellular internalization, efficiently loading into EVs upon co-incubation without altering vesicle size distribution or integrity [119,120,121].

In addition to cationic polymers, peptides binding to EVMs are also important in nucleic acid drug delivery research. Cell-penetrating peptides (CPP) are rich in cationic amino acids such as arginine (R) and lysine (K), which are frequently used in gene delivery [122,123]. Some peptide motifs can bind with nucleic acids to facilitate highly efficient loading into EVMs. Ghulam et al. [124] observed that fusing the glycolytic enzyme glyceraldehyde-3-phosphate dehydrogenase (GAPDH)-derived G58 peptide to dsRNA-binding motifs enabled the highly efficient loading of small interfering RNA (siRNA) onto the EV surface, resulting in efficient delivery of siRNA to multiple anatomical regions of the brain. Shabanali et al. [125] transduced a lentiviral vector bearing the LAMP2b-DARPin G3 chimeric gene into HEK293T cells and successfully delivered siRNA against the TPD52 gene into HER2-positive breast cancer cells through targeted EVs bearing the Lamp2B-DARPin chimeric protein, modulating TPD52 gene expression. These approaches have the potential to facilitate gene transfer to target cells, providing an additional option for gene therapy and drug delivery.

### 3.3. EVM Delivery of Proteins/Peptides

The use of biological functional enzymes and therapeutic proteins in clinical and pre-clinical treatments can help inhibit the occurrence and progression of tumors. However, there are still several challenges in the process of systemic protein administration to treat tumor diseases, including easy degradation, low bioavailability, poor targeting, and short half-life. Engineered EVs are ideal candidates for delivering therapeutic proteins, receptors, ligands, cytokines, and monoclonal antibodies as they can be effectively used for tumor targeting. Hao et al. [126] reported that lentiviruses expressing Tet-sFlt-1 infected HEK293 cells to obtain soluble fms-like tyrosine kinase-1 (sFlt-1)-enriched EVs. The EVMs suppressed the growth of small-cell lung cancer by inhibiting endothelial cell migration.

Recently, the crucial advantages of EVs have been investigated as a delivery system for membrane protein therapeutics. Compared with ferritin nanocages, signal regulatory protein α (an antagonist of CD47 on tumor cells) expressed on EVMs is more conducive to the spatial arrangement of binding with CD47 on the surface of tumor cells. This makes the protein-loaded EVMs capable of further enhancing the phagocytosis of bone marrow-derived macrophages to tumor cells and subsequently inhibiting tumor growth in vivo [127].

In addition to pre-loading proteins into EVs, other post-loading methods such as sonication, freeze–thaw, and saponin have been studied. Haney et al. [101] found that EVs prepared by sonication and saponin had the highest loading efficiency and release of active catalase. Lysosome-associated membrane protein 2 specifically binds to the acetylcholine receptor, allowing for the targeting of neurons, oligodendrocytes, and microglia [61]. Ye et al. [128] reported a simple and versatile approach to functionalizing drug-loaded EVs with the pro-apoptotic peptide KLA and the targeted peptide, low-density lipoprotein (LDL), which selectively binds to the LDL receptor (LDLR) overexpressed on the blood–brain barrier (BBB) and the glioblastoma multiforme (GBM) cell lines. This resulted in an increased survival rate in mouse models of glioma.

**Table 2:** Most recent advances of polymers in engineering EVMs for cancer treatment.

Type of Drugs	Type of EVM	Cargo	Polymer	Application	Conclusion	Ref.
small molecule chemotherapeutics	hybrid EVs	ICG, R837	CD47	Drug delivery: long circulation and excellent photothermal therapy	CD47-overexpressed hybrid therapeutic nanovesicles exhibited long blood circulation and improved the macrophage-mediated phagocytosis of tumor cells.	[84]
	Sur-EVMs	doxorubicin	Ang and TAT	Delivery efficiency: targeting	Cross the BBB, reach the glioma, and penetrate the tumor.	[109]
	Syn-EVMs	gemcitabine	PEG	Drug delivery for endosomal escape and long circulation	Accelerate drug release at endosomal level, and without significantly compromising their stealth abilities.	[129]
nucleic acid	hybrid EVs	CRISPR/Cas9	Lipofectamine 2000	CRISPR/Cas9 system delivery for in vivo gene editing	Systems require delivery of both Cas9 and sgRNA into the same cell, which remains a challenge in vivo where delivery and editing efficiency remain low.	[82]
	Sur-EVMs	siRNA	G58 peptide	Gene delivery: siRNA loading	GAPDH-derived G58 peptide enables highly efficient loading of siRNA onto the EV surface	[124]
	Syn-EVMs	siRNA	PEI	Gene delivery: loading and responsive release	Photoactivatable polymer permitted the efficient loading and ROS-responsive release of siRNA.	[130]
proteins/ peptides	hybrid EVs	CRISPR associated protein 9	dioctadecyl-amido-glycyl-spermine	Protein delivery: a simple and quick method for loading proteins in EVs	EV-mediated delivery is compatible with various molecular weight proteins and improved uptake compared to electroporation.	[131]
	Sur-EVMs	sgRNA and Cas9 protein	GFP antibody	A method to deliver sgRNA: Cas9 ribonucleoprotein	This engineered a modified exosome-fused CD63 with GFP, which can bind to the GFP antibody fused with Cas9 protein. Cas9 proteins were captured and efficiently loaded into exosomes rather than a random package.	[132]
	Sur-EVMs	proapoptotic peptide and methotrexate	low-density lipoprotein, KLA peptides	Peptide delivery: for brain tumor treatment	Functionalizing chemotherapeutics-loaded EVs with peptides in a facile and controllable way facilitates them targeting BBB and brain tumor cells with highly efficient anticancer properties.	[128]

## 4. The Challenges and Perspectives of EVMs for Drug Delivery

Since the discovery of EVs as natural mediators for intercellular communication, these nanoscale vesicles have been extensively researched as a potential solution to mitigate problems faced by conventional drug delivery systems. However, current studies on EVM-based drug delivery systems are still in their infancy. A deeper understanding of the key surface cues on EVs responsible for intrinsic targeting and pharmacological effects, as well as advanced technologies to isolate EVs in good yield and high purity, are necessary before these systems can be extensively applied as nanomedicines for cancer treatment. Better drug delivery strategies should also be developed to optimize the therapeutic efficacy of EVM-based drug delivery systems.

Natural polymers, such as proteins and aptamers, have been widely used to better imitate [133,134] and optimize targeting functions [135,136]. Synthetic polymers such as PEG, PEI, and pCBMA can be utilized for more efficient delivery [91,116,117,137]. These applications for improved drug delivery strategies are described in Section 2 and Section 3. However, production and purification methods also restrict the application and translation of EVMs. Therefore, strategies for further improvement should be considered.

**Improving the efficiency of production.** Various approaches have been developed to increase EV secretion rates and improve production yield [138]. EV mass quantification revealed a several-fold increase in EV secretion using 3D cultivation methods compared to 2D monolayers [139,140,141]. The hypoxia, cell density, and non-adherent cell morphology within the 3D spheroids may be causative factors affecting cell viability, thereby altering EV synthesis and secretion [142,143,144,145]. Studies have demonstrated the superior cytocompatibility of zwitterionic biomaterials compared to other commonly used biomaterials for the preparation of hydrogels, such as poly(hydroxyethyl methacrylate) [146,147,148]. Zwitterionic hydrogels significantly outperformed other culture systems for embryonic cells, cancer cells, and stem cells due to their hydrophilicity and resistance to nonspecific protein adsorption, resulting in prolonged cell longevity and sustained stem cell multipotency [149,150]. The capacity for maintaining cell viability based on zwitterionic hydrogels may provide a better platform for EV production by a top-down approach.

**Figure 3:**
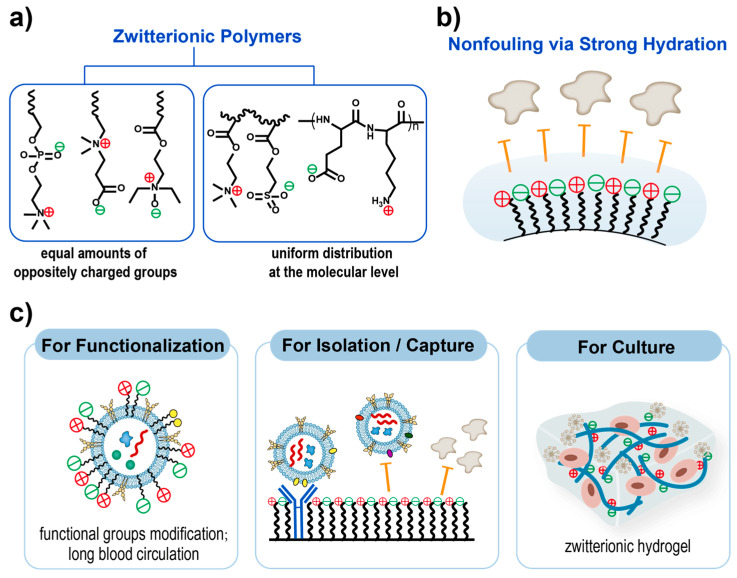
Zwitterionic materials applied in extracellular vesicles mimetics. (**a**) The classification of zwitterionic polymers. The two key criteria are as follows: equal amounts of oppositely charged groups and uniform distribution at the molecular level (minimized dipole). (**b**) Illustration of the key nonfouling mechanisms of zwitterionic materials. (**c**) Applications of zwitterionic materials in engineering extracellular vesicles. The left box shows the functionalization of EVMs by zwitterionic polymers; the middle box exhibits the application of zwitterionic modified materials for the isolation or capture of certain EVs without specific protein adsorption; the right box illustrated the application of zwitterionic hydrogel for cell culture to obtained EVs.

**Improving the efficiency of purification.** Several methods, such as centrifugation [151,152,153], ultrafiltration [154,155], size exclusion chromatography [131,155,156], and microfluidics [157,158,159], can be used to isolate EVs, which determine the sample yield and purity for further application. Precipitation is a feasible approach to separate target EVMs with a less damaging effect on EVs, which can maintain more natural EV characteristics [160]. Hydrophilic polymers, such as PEG, can wrap dozens or hundreds of EVs together by reducing solubility, forming extracellular vesicle aggregates that can be easily precipitated by low-speed centrifugation [161,162,163]. Various commercial kits based on polymer precipitation methods are now available, such as ExoQuick™ (System Bioscience, Palo Alto, CA, USA) and Exo-spin™ (Cell Guidance Systems, Cambridge, UK) [160,164]. In addition, polymers, especially polyelectrolytes such as cationic poly(L-lysine) (PLL), can aggregate anionic EVs due to charge interaction, thereby facilitating separation and providing a new idea for EVM separation [165]. Furthermore, zwitterionic biomaterials could be developed for diagnostic applications on EVs due to their ability to resist nonspecific protein adsorption. As early as 2012, researchers developed polymer-coated immunoaffinity beads modified with sulfobetaine moieties to capture EVs in serum [166]. Yoshida et al. [167] synthesized an EpCAM-affinity coating agent consisting of a peptide aptamer for EpCAM and a zwitterionic poly-2-methacryloyloxyethyl phosphorylcholine polymer, allowing the concentration of cancer-related EVs from heterogeneous EV mixtures. Some studies found that materials based on zwitterionic polymers captured amounts of exosomal protein comparable to a commercial EV isolation kit [168]. These studies have proved that zwitterionic biomaterials can be used for the capture or isolation of extracellular vesicles, which is a key process for understanding and applying extracellular vesicle mimetics (Figure 3).

**Further development of clinical trials.** Compared to liposomes, which have been widely used in cancer therapy and clinical trials [169], natural-EV based EVMs are still in the preliminary stages of clinical research. Codiak’s exoSTING (engineered exosomes containing small-molecule stimulator of interferon genes (STING) agonist) and exoIL-12 (engineered exosomes containing IL-12) were launched in phase I clinical trials for immune activation in cancer. MD Anderson has initiated a phase I clinical trial of Kalluri’s RAS^G12D^ siRNA-containing exosomes for improving survival in pancreatic cancer [170]. If Codiak’s EV-based therapy proves safe and reliable, the next question is whether EV-based drug delivery systems have a greater advantage than existing systems. To this end, more characterization and efficacy evaluation methods need to be updated and iterated. For example, 3D printing technology could be used to establish 3D tumor models for drug evaluation [171].

## 5. Conclusions

Extracellular vesicle mimetics offer new insights into the utilization of biomimetic nanotechnology to potentiate the treatment of cancers by small molecule chemotherapeutics, nucleic acids, and proteins. This review has summarized the characteristics of extracellular vesicles based on their structure, and introduced structure engineering by polymers to meet the requirements of drug delivery based on different preparations. We have highlighted the application of polymers in EV-based cancer therapy mainly for prolonging circulation and improving targetability. Although the emergence of EVMs can overcome some obstacles set by natural EVs properties, there are still challenges that need to be investigated, such as a deeper understanding of biological systems, more advanced biomimetic nanotechnology, and more efficient production. Lastly, we look forward to the development prospects of EVMs in cancer drug delivery, especially for polymer-based strategies on production and purification. As more attention is paid to the application of EVMs in various fields, we believe that more effective strengthening strategies will be developed, ultimately moving towards practical applications.

## Data Availability

Not applicable.
